# Evaluation of Methods to Estimate Understory Fruit Biomass

**DOI:** 10.1371/journal.pone.0096898

**Published:** 2014-05-12

**Authors:** Marcus A. Lashley, Jeffrey R. Thompson, M. Colter Chitwood, Christopher S. DePerno, Christopher E. Moorman

**Affiliations:** 1 Department of Forestry and Environmental Resources, North Carolina State University, Raleigh, North Carolina, United States of America; 2 SAS Institute Inc., Cary, North Carolina, United States of America; University of Marburg, Germany

## Abstract

Fleshy fruit is consumed by many wildlife species and is a critical component of forest ecosystems. Because fruit production may change quickly during forest succession, frequent monitoring of fruit biomass may be needed to better understand shifts in wildlife habitat quality. Yet, designing a fruit sampling protocol that is executable on a frequent basis may be difficult, and knowledge of accuracy within monitoring protocols is lacking. We evaluated the accuracy and efficiency of 3 methods to estimate understory fruit biomass (Fruit Count, Stem Density, and Plant Coverage). The Fruit Count method requires visual counts of fruit to estimate fruit biomass. The Stem Density method uses counts of all stems of fruit producing species to estimate fruit biomass. The Plant Coverage method uses land coverage of fruit producing species to estimate fruit biomass. Using linear regression models under a censored-normal distribution, we determined the Fruit Count and Stem Density methods could accurately estimate fruit biomass; however, when comparing AIC values between models, the Fruit Count method was the superior method for estimating fruit biomass. After determining that Fruit Count was the superior method to accurately estimate fruit biomass, we conducted additional analyses to determine the sampling intensity (i.e., percentage of area) necessary to accurately estimate fruit biomass. The Fruit Count method accurately estimated fruit biomass at a 0.8% sampling intensity. In some cases, sampling 0.8% of an area may not be feasible. In these cases, we suggest sampling understory fruit production with the Fruit Count method at the greatest feasible sampling intensity, which could be valuable to assess annual fluctuations in fruit production.

## Introduction

Because plant regeneration is strongly dependent upon seed dissemination, many plants have adapted fruits with edible and nutritive fleshy pulps to encourage consumption by animals for seed dispersal [1]. Seeds of some fruit-bearing flora have adapted mechanisms to avoid damage from digestive enzymes and may even require scarification for germination [2]. Concomitantly, mammals, birds, and reptiles have adapted dietary niches to consume fleshy fruits (also referred to as soft mast; hereafter fruit) as a primary or supplementary energy source [3], leading to a dynamic relationship between seed dispersers and respective fruit-bearing flora [1].

Measuring fruit biomass may be important for evaluating a variety of research questions. For example, estimating fruit biomass may be useful to evaluate mechanisms regulating animal populations, particularly frugivores [4]. Also, fruit biomass may explain variations in animal behavior and could be used to evaluate the potential effects of forests silvicultural practices on fruit consumers, plant dissemination, plant regeneration, and conservation of plant communities [1]. When used to address research hypotheses, accurate estimates of fruit biomass will be needed. Furthermore, an accurate and practical method for estimating fruit production may be necessary for land managers to monitor fruiting responses to management regimes because fruit abundance may change rapidly with disturbance and forest succession [4]–[13].

A variety of protocols to monitor fruit production have been developed, with the majority focusing on estimating fruit biomass in the forest canopy [14]–[16]. Visual estimations and fruit traps commonly are used to evaluate fruit biomass in the overstory [14], [16]–[21]. However, Parrado-Rosselli et al. [15] reported fruit traps were ineffective at monitoring fruit production in tropical forest canopies and suggested observing fruit in the overstory along systematically located transects was the only time-efficient way to monitor overstory fruit production [22]. Plant coverage and visual estimations have been used to monitor understory fruit production [9], [10], [13], [23]. Some studies estimated understory fruit availability in terms of percent land cover of fruit-producing plants [24], [25], but plant cover may not be a reliable measure of fruit production [8]. Though visual estimation or collection of fruits has been used to measure understory fruit availability in many studies, the reported protocols were inconsistent [9], [10], [13], [23].

The problems with current methods are two-fold: 1) protocols are not standardized; and 2) little is known about the relative accuracy and time efficiency of each. Accuracy and feasibility contribute equally to the adequacy of the methods used to quantify fruit biomass. Therefore, a comparative evaluation of both the accuracy and practicality among protocols is needed. We compared 3 methods of estimating understory fruit availability. Our study was designed to test each method in 3 measures of utility listed in order of importance:

1. Estimation ability

2. Sampling intensity necessary for estimation ability

3. Time commitment to conduct each method

## Materials and Methods

### Ethics Statement

This research was performed in accordance with the United States Department of Defense and Fort Bragg Military Installation. No animals were handled in this study. No permits were required for the described study, which complied with all relevant regulations.

### Site Description

We conducted our study at Fort Bragg Military Installation (∼65,000 ha, hereafter Fort Bragg), located in the Sandhills physiographic region in southeastern North Carolina, USA (358170N, 828470W), during July 2011. Roughly 65% of Fort Bragg was forested, which was second and third growth longleaf pine (*Pinus palustris*; [26]). Dominant understory fruit producing plant species included blueberries (*Vaccinium* spp.), huckleberries (*Gaylussacia* spp.), gallberries (*Ilex* spp.), grapes (*Vitis* spp.), Atlantic poison oak (*Toxicodendron pubescens*), greenbriers (*Smilax* spp.), and blackberries (*Rubus* spp.). Fort Bragg was managed by the United States Department of Defense under a 3-yr fire-return interval, with prescribed burning primarily during the growing season. Timber was managed on a 120-year rotation for longleaf pine and a 100-year rotation for other pines [26], [27].

### Study Design

**Figure 1:**
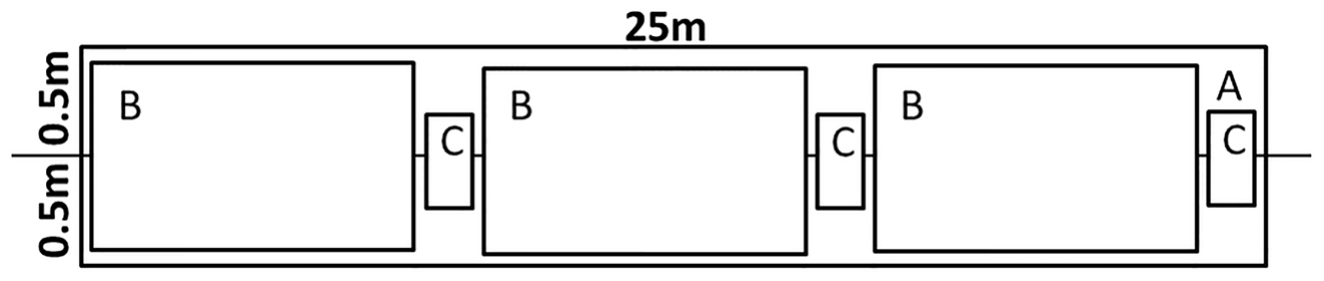
Diagram of sampling scheme for evaluating 3 methods of estimating fruit production. Plot A representing entire 25-m^2^ sampling area and Plots B (3 m^2^) and C (0.1 m^2^) nested within. Note: Plots A, B, and C in the diagram are not drawn to scale for ease of display

In 2011, we randomly established 30 25-m transects in each of 4 cover types (upland hardwood, hereafter UH; bottomland hardwood, hereafter BH; upland pine following dormant-season fire, hereafter DSUP; and upland pine following growing-season fire, hereafter GSUP) (n = 120). We systematically centered 7 different sampling plots on each transect (Figure 1). Plot A comprised the entire area along the transect, 1 m wide and 25 m long. Within Plot A, 3 Plots B (3-m^2^ each) were placed at 5, 12.5, and 20 m along the transect, and 3 Plots C (0.1-m^2^ each) were placed at 7.5, 15, and 22.5 meters along the transect.

We used 3 methods to estimate fruit biomass: Fruit Count, Stem Count, and Plant Coverage. We counted fruits, stems, and coverage of blackberries, huckleberries, blueberries, and gallberries. We chose these genera based on plant fruiting phenology, wide occurrence across the temperate zone, high wildlife food value, and frequent occurrence at Fort Bragg [28].

### Fruit Count

We used methods outlined in Jackson et al. [23] to guide the Fruit Count protocol. Jackson et al. [23] collected fruits in 2-m-wide plots along 50-m transects. We modified the protocol by shortening the transects from 50 m to 25 m, decreasing width from 2 m to 1 m, and counted fruits rather than collecting fruits, which decreased our time commitment per plot. We modified the methods used by Jackson et al. [23] because the additional time commitment associated with collecting, drying, and weighing fruits is not practical in most cases. Furthermore, the purpose of our proposed Fruit Count method was to evaluate whether a more time efficient visual count of fruit could accurately estimate the actual fruit biomass present. We counted fruits within 0.5 m along each side of the 25-m transect (Plot A, Figure 1), noting the number of fruits occurring in Plots B and C to test required sampling intensity to accurately estimate fruit production (see *Sampling Intensity* section). Observers counted fruits at a walking pace with minimal pause for counting to reduce time per transect. Therefore, fruit counts are not absolute counts but the best estimate of the number of fruits present.

### Stem Count

We used methods outlined in previous literature to guide the stem count protocol [21], [29], [30]. These include 3 systematically located 3-m^2^ plots along a 50-m transect. Originally, the Stem Count method was designed to measure diet selection in herbivores, so we modified the method by counting only fruit producing plants. We counted stems of fruit producers only within Plot B (Figure 1). The plot size of plot B was chosen because larger plots may inflate observer error and result in an unreasonable time commitment per plot.

### Plant Coverage

We used methods outlined in Daubenmire [31] to guide the plant coverage protocol. Daubenmire [31] used a 0.1-m^2^ frame to estimate plant coverage. The original method was designed to sample ground cover, but we modified the method to measure cover of fruit bearing species. We estimated plant coverage of fruit producers within Plot C (Figure 1). We only assessed plant coverage in Plot C because plot sizes greater than 0.1 m^2^ may increase observer error [31]. The same 2 observers were used for all plots to reduce bias.

### Absolute Biomass

After counts from all three methods were recorded, we collected all fruits within Plot A (i.e., total area surveyed). We kept fruits from each respective plot type separate, which yielded 7 bags (e.g., 1 bag for plot A excluding plots B and C, 3 bags for plot B, and 3 bags for plot C). The absolute fruit biomass (total fruits in plot A) was the sum of all bags collected. We dried fruits in a convection oven at 50 °C for 48 hours and weighed them with seeds (by species) to the nearest 0.01 g keeping biomass from each bag separate. The fruits were not collected as part of the protocol for the three methods; rather, they only were collected so we could compare the accuracy of each method in estimating the actual biomass present.

### Testing Accuracy

We used each method to estimate absolute fruit biomass for the respective sampled area (e.g., plant coverage estimated absolute fruit biomass for Plot C). We used fruit biomass as the response variable rather than fruit count, but the same results should be expected with either metric (see Greenberg et al. [13]; Figure 1). Likewise, fruit biomass and fruit count would have yielded identical results in our study because fruit weights were all 0.05 g. We chose a continuous distribution of responses and analyzed absolute fruit biomass under the assumption of normality. Initially, we ran a simple correlation to determine if fruit counts, stem density, and Daubenmire scores were correlated to fruit biomass. Then, to determine if a method could predict fruit biomass, we estimated each model using the QLIM (qualitative and limited dependent variable model) procedure, which is the appropriate SAS procedure to perform with censored distributions for a continuous response variable [32]. This procedure is part of the SAS/ETS (econometric, time series) procedures. The linear regression model is as follows: Y* = β_1_X + β_2_I_BH_ + β_3_I_DSUP_ + β_4_I_GSUP_ + β_5_I_UH_ + ε*, where Y  =  biomass of berries, *X*  =  measurement method, *I_BH_* is an indicator of specific cover type (so it equals 1 if cover type is BH and equals 0 otherwise), the other indicator variables are defined in a similar way for their respective cover types, and *ε* is a normally distributed error with mean 0. We used a censored normal distribution for the response Y (fruit biomass). This means fruit biomass in an area of forest follows a normal distribution but is censored at 0, so fruit biomass cannot be less than 0. The assumption of normality was verified by looking at histograms for each measurement method. We set alpha = 0.05. A non-significant result (large P-value associated with the model parameter which leads to not rejecting the null hypothesis of *β*_1_ = 0) indicated the method was not useful in predicting fruit biomass. A significant result (small P-value rejecting the null hypothesis of *β*_1_ = 0) indicated the measurement method was useful for predicting fruit biomass.

Then, we used the Akaike information criterion (AIC) as a measure of the relative “goodness of fit” of each significant statistical model [33]. The AIC is based on the concept of information entropy, in effect offering a relative measure of the information lost when a given model is used to describe reality. Furthermore, we used AIC which was corrected for finite sample sizes (AICc) as a secondary measure of model fit to corroborate the AIC model ranking [34]. Burnham and Anderson [34] recommended using AICc, rather than AIC, if *n* is small or *k* (the number of parameters in the model) is large because it gives more penalty to small sample sizes and additional parameters. However, AICc ranked the competing models identically to that of AIC and therefore was not reported.

### Sampling Intensity

We estimated the fruit biomass of Plot A with Fruit Counts from various combinations of Plots B and C (i.e., all C plots equal 1.5-m^2^ area or 1.2% sampling intensity) to test the effectiveness of the Fruit Count method at 0.4%, 0.8%,1.2%, 4%, 8%, and 12% sampling intensities. At each sampling intensity, we used QLIM procedure as before to test the accuracy of the Fruit Count method. We used a Bonferroni correction to control the overall Type 1 error rate. We did not test sampling intensity of Stem Count or Plant Coverage methods because the Fruit Count method was the superior measurement method based on results of the accuracy test described above.

## Results

Fruit Count (R^2^ = 0.90, P<0.001) and Stem Density (R^2^ = 0.18, P<0.001) were correlated with fruit biomass, indicating both methods were potentially useful even though Fruit Count was substantially more correlated than Stem Density. However, Plant Coverage (R^2^<0.001, P = 0.82) was not correlated to fruit biomass. Fruit Count and Stem Density could be used to accurately estimate fruit biomass at the 100% sampling intensity (Table 1). Plant Coverage did not have significant predictive value at a 100% sampling intensity (P = 0.21).

**Table 1:** Effectiveness of Fruit Count, Plant Coverage, and Stem Count methods at predicting understory fruit biomass at varying sampling intensities on Fort Bragg Military Installation, NC (July 2011).

	100% Sample		12% Sample		8% Sample		4% Sample		1.2% Sample	0.8% Sample	0.4% Sample
Method	P-Value	AIC	P-Value	AIC	P-Value	AIC	P-Value	AIC	P-Value	P-Value	P-Value
Fruit Count^a^	<0.0001^b^	51.99	<0.0001	289.89	<0.0001	304.92	0.0008	313.04	<0.0001	0.0024	0.2437
Stem Count^a^	<0.0001	197.28	<0.0001	308.57	0.0015	313.98	0.0066	316.8	N/A	N/A	N/A
Plant Coverage	0.2090	N/A									

a Fruit Count and Stem Count were extended to smaller sampling intensities for comparison

b The P-value is testing whether the method accurately estimated the fruit biomass for the sampled area. A Bonferroni correction was used and P-values are significant if P≤0.008.

Fruit Count and Stem Count were viable predictors of fruit biomass when sampling as little as 4% of the area and Fruit Count was useful even as small as 0.8% of the area (Table 1). However, Fruit Count was superior to the Stem Count at all sampling levels in terms of P-value magnitude and AIC scores (Table 1). Also, the Fruit Count method (9 ±0.5 seconds/m^2^) was more time efficient than the Stem Count method (18 ±1 seconds/m^2^), requiring half the time commitment for sampling.

## Discussion

We showed Plant Coverage was not a viable method of estimating understory fruit biomass, likely because of variations in fruit production annually and among individual plant or plant species [13]. Although the Stem Density method could be flawed for the same reason, Stem Density may have shown more promise because it takes into account the number of fruit producing plant stems rather than the coverage of fruit producing plants. Conversely, the Fruit Count method worked better than the other 2 methods because fruiting phenology, genetics, and environmental influences on an individual plant's fruit production do not inflate error when estimating fruit availability. Although the Fruit Count method is affected by spatio-temporal variability in fruit biomass, increasing sampling intensity can improve the precision of estimates.

Because sampling at least 0.8% of the land area may be required to accurately estimate fruit biomass, quantification of fruit production on larger landholdings may not be feasible. For instance, Fort Bragg includes about 42,000 forested ha. To accurately estimate fruit production for the entire site, sampling would have to cover 336 ha, which is not practical. However, where fruit availability is more homogeneous, smaller portions of the landscape may be sampled to accurately estimate fruit biomass, which may be more practical particularly if a set amount of time is allotted per transect [35]. Additionally, the use of permanent transects to sample yearly may allow managers to monitor relative rather than absolute fruit biomass; this strategy would allow managers to track the direction of change in fruit production following management prescriptions using lower sampling intensity, though sampling would still be required yearly unless habitat characteristics remained similar. Finally, sampling efforts could be framed on a smaller scale (e.g., vegetation type) to focus inferences where a plant species and associated fruit production are more abundant or consistent.

Researchers and practitioners must consider their objectives to determine whether estimating fruit biomass is necessary. For example, if an experiment was designed to evaluate the effects of silvicultural treatments on fruit production, estimating fruit abundance may be useful to address the hypotheses. However, in many cases researchers and practitioners may be able to effectively address questions or monitor management practices by measuring relative changes in fruit production rather than estimating actual fruit biomass. Furthermore, only presence or absence data over multiple years may be sufficient to guide timber harvest recommendations in some cases [36]. For example, Lashley et al. [36] demonstrated monitoring the presence or absence of mast on trees in the overstory could effectively identify target trees to retain while harvesting timber. In any case, we recommend the Fruit Count method over other methods to monitor understory fruit biomass because the estimates from this method are more robust to variations in fruit densities and the method requires relatively less time to conduct surveys.

## Conclusions

Fruits are an important component in the diets of many wildlife species. Monitoring shifts in fruit biomass is important to ensure fruit production is maintained when managing plant communities for wildlife. The Fruit Count method provides an easy and repeatable protocol that allows researchers and land managers to measure fruit biomass with a small relative time commitment, while maintaining accurate estimates that are comparable across studies and years within a study. We recommend using the Fruit Count method at the greatest feasible sampling intensity to estimate fruit production. When target sampling intensities are not feasible, use of the Fruit Count method with permanent plots to evaluate trends in year-to-year fruit biomass may suffice.
